# Development and evaluation of a machine learning-based risk prediction model for enteral feeding intolerance in sepsis patients

**DOI:** 10.3389/fnut.2026.1858783

**Published:** 2026-06-10

**Authors:** Zhengang Wei, Congcong Liu, Jicheng Zhang, Xiaohua Wang

**Affiliations:** 1 Department of Critical Care Medicine, Shandong Provincial Hospital Affiliated to Shandong First Medical University, Jinan, China; 2 Department of Information Technology, Affiliated Hospital of Zunyi Medical University, Zunyi, China

**Keywords:** enteral feeding intolerance, enteral nutrition, machine learning, predictive model, sepsis

## Abstract

**Background:**

Early detection and prediction of enteral feeding intolerance (EFI) are essential for effective management of septic patients. This study seeks to develop an interpretable machine learning (ML) model for predicting EFI in septic patients.

**Methods:**

Data were collected from septic patients admitted to the intensive care unit and receiving enteral nutrition (EN) at a tertiary hospital in Shandong Province between January 2023 and July 2025. A retrospective cohort was randomly divided into training and validation sets in a 7:3 ratio. Feature selection was performed using univariate analysis and binary logistic regression. 5 independent ML models were developed and evaluated based on the area under the receiver operating characteristic curve (AUROC), accuracy, sensitivity, specificity, and F1 score. The Shapley Additive Explanation (SHAP) method was applied to interpret the predictive model.

**Results:**

The study included 549 septic patients, with an EFI incidence of 34.6%. 6 key features were selected for model development: age, APACHE II score, albumin levels, receipt of continuous renal replacement therapy, EN start time, and intra-abdominal pressure. Among the models, the RF model demonstrated the best performance, with an AUROC of 0.891, accuracy of 0.830, F1 score of 0.771, specificity of 0.869, and sensitivity of 0.763. SHAP analysis identified albumin levels as a protective factor for EFI in septic patients.

**Conclusion:**

This model can serve as a tool to identify high-risk individuals with EFI among septic patients, facilitating clinical healthcare providers in delivering scientific and individualized EN therapy to patients.

## 1 Introduction

Sepsis is a rapidly progressing condition, characterized by organ dysfunction due to a dysregulated host response to infection, and is associated with a high mortality rate, making it one of the leading causes of death among infectious diseases globally (1). A recent systematic review and meta-analysis found that the hospital incidence of sepsis globally is approximately 189 cases per 100,000 person-years, with a mortality rate as high as 26.7% (2). Additionally, a cross-sectional study conducted across 44 hospitals in China showed that sepsis affects one-fifth of intensive care unit (ICU) patients, with a 90-day mortality rate of 35.5% (3). In response, the World Health Organization has classified sepsis as a major public health issue and actively advocates for the optimization of prevention, diagnosis, and management to alleviate the burden of sepsis (4). The pathogenesis of sepsis is complex, and the gastrointestinal system is one of the most commonly affected organs, with patients often experiencing nutritional and metabolic changes, gut microbiota imbalance, and immune dysregulation (5). Studies have shown that the risk of malnutrition in critically ill patients is approximately 55.09%, with sepsis patients having a risk of 10.0 to 20.0% (6, 7). On the one hand, excessive inflammatory response can lead to gastrointestinal dysfunction and poor nutrient absorption, further exacerbating the nutritional status of sepsis patients (8). Therefore, during the treatment of sepsis, nutritional support therapy is crucial for patient recovery and prognosis.

Enteral nutrition (EN), as a core treatment for sepsis, has been shown to improve immune imbalance and reduce intestinal barrier damage (9). However, due to the stress response in sepsis patients, along with gastrointestinal ischemia, dysfunction, and mucosal malnutrition, gut microbiota translocation often occurs. This can activate adenylyl cyclase in intestinal epithelial cells, inhibiting the absorption of water and sodium, while promoting chloride secretion, leading to the development of enteral feeding intolerance (EFI) (10). EFI is a common complication of EN, triggered by multiple factors such as high gastric residual volumes, nausea/vomiting, bloating, and diarrhea, which leads to a reduction in EN intake, preventing patients from reaching the target of 20 kcal/(kg·d) within 72 h (11). Studies have shown that the incidence of EFI in sepsis patients during EN ranges from 25.0 to 41.27% (12). Once EFI occurs, clinicians often reduce or discontinue nutritional support, increasing the risk of malnutrition and resulting in failure to meet prescribed target feeding volumes. In addition, malnutrition is associated with higher complication rates, prolonged hospital stays, and increased mortality (13). Therefore, early identification of the risk of EFI in sepsis patients, along with the effective implementation of clinical and nutritional management, is a critical issue in intensive care medicine that requires urgent attention and research.

The occurrence of EFI in sepsis patients is influenced by various factors, including the patient’s condition and the treatment and care provided. Existing research has mainly focused on analyzing susceptibility factors. Although some EFI prediction models have been developed, these models often rely on traditional logistic regression or nomograms, incorporating a limited number of risk factors, and are primarily aimed at ICU patients rather than focusing specifically on sepsis patients (14–16). As an important subset of artificial intelligence, machine learning (ML) has shown better predictive performance in modeling complex and nonlinear effects in the medical field (17). Based on this, the present study intends to use a retrospective cohort design to construct and validate a risk prediction model for EFI in sepsis patients using ML algorithms. The goal is to identify high-risk individuals for EFI, enable early intervention, and ultimately improve the target calorie intake rate and patient outcomes.

## 2 Methods

### 2.1 Study population

**Figure 1:**
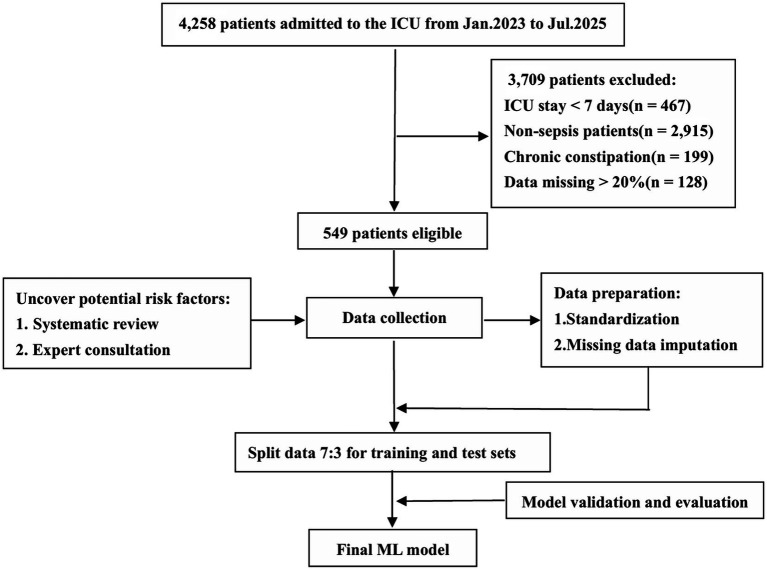
Flowchart of patient enrollment. This flowchart illustrates the enrollment and grouping process of 549 septic patients in this study.

This retrospective cohort study collected data from electronic and digital medical record systems. A total of 549 patients admitted to the ICU of a tertiary hospital in Shandong Province between January 2023 and July 2025 were enrolled. The study design details are presented in Figure 1. Inclusion criteria were as follows: (1) age ≥ 18 years; (2) meeting the Sepsis 3.0 diagnostic criteria (18); (3) ICU stay ≥7 days; and (4) receiving EN support after ICU admission. Exclusion criteria included: (1) discontinuation of EN for reasons unrelated to feeding intolerance (e.g., surgery or specific treatments); (2) presence of gastrointestinal malignancy, chronic diarrhea, or gastrointestinal bleeding; and (3) missing data exceeding 20%. This study was approved by the Institutional Ethics Committee (code: SWYX:2025–239).

### 2.2 Assessment of EFI

The criteria for EFI were defined based on the guidelines established by the European Society of Intensive Care Medicine (ESICM) Abdominal Problems Working Group (WGAP) (11), with adjustments made to reflect clinical practice. EFI was defined as meeting any one of the following criteria: (1) Inability to meet the target caloric intake of at least 20 kcal/kg·d within 72 h after initiating EN; (2) The occurrence of symptoms such as bloating, vomiting, or diarrhea during EN, leading to the suspension or discontinuation of enteral feeding; (3) Gastric residual volume (GRV) ≥ 200 mL at any single measurement or total GRV exceeding 500 mL within 24 h, measured every 6 h. In this study, the occurrence of EFI was defined as meeting any one of the above criteria.

### 2.3 Variable collection

This study identified predictive factors based on a review of existing literature and expert input, comprising 29 risk factors categorized into 3 main groups: sociodemographic data, disease-related factors, and laboratory biochemical markers, including complete blood count and blood gas analysis. To ensure consistent results, the retrospective cohort dataset was collected by 2 uniformly trained researchers using standardized Excel spreadsheets. The worst values of the patient’s scores and laboratory indicators during the first 24 h after admission were recorded. EFI occurrence was monitored from the initiation of EN over a 7-day observation period. Both researchers were proficient in using the electronic medical record system and adhered to privacy protection guidelines during data collection. The complete dataset was securely stored by a designated individual, and all data underwent dual verification and cross-checking by the 2 researchers, with random checks performed by 1 third researcher to ensure accuracy and reliability.

### 2.4 Data preprocessing

Data cleaning and preprocessing involved standardisation, converting textual descriptions into numerical values to ensure dataset quality and accuracy. Binary variables (e.g., gender) were encoded as follows: female = 0, male = 1. Patients diagnosed with EFI were considered “cases,” and those without EFI were classified as “controls,” encoded as 1 and 0, respectively. Missing values were imputed using the Expectation–Maximization method for continuous variables and the mode for categorical variables. Patients with more than 20% missing data were excluded. Additionally, the sample data were normalized. Data preprocessing helped maintain the integrity of the original data and improve the efficiency of data mining (19). Data analysis was conducted using IBM SPSS Statistics version 29.0 (IBM Inc., Armonk, NY, USA). Descriptive data, such as sociodemographic variables and disease-related factors, were presented as frequencies and percentages. The chi-square (*χ*^2^) test was used to compare group differences. Non-normally distributed data, such as laboratory biochemical markers, were described using median and interquartile range [M (P25, P75)]. The rank-sum test was used for analysis. All statistical analyses were two-tailed, with a *p*-value of less than 0.05 considered statistically significant.

### 2.5 Model data selection

Effective feature selection is critical in predictive modeling, as too many variables may lead to overfitting and reduced computational efficiency, while too few variables may miss important patterns (18). We first selected features through univariate analysis and then refined the predictors using logistic regression, resulting in risk factor indicators and data. The retrospective cohort data was randomly split once to maintain sample balance, with 70% (384/549) used for model training and 30% (165/549) used for internal validation. 5 independent ML algorithms were employed to develop the EFI prediction model for septic patients.

### 2.6 Independent ML model development

5 independent ML techniques, including Random Forest (RF), Support Vector Machine (SVM), Neural Networks, XGBoost, and SVM algorithms, were implemented in Python (version 3.8). To mitigate overfitting, grid search with 10-fold cross-validation was applied for hyperparameter tuning on the training dataset. The model’s authenticity and predictive power were evaluated using a confusion matrix and the area under the receiver operating characteristic curve (AUROC), specifically assessing performance through AUROC, accuracy, specificity, sensitivity, and F1 score. Additionally, we used the SHAP method to interpret and visualize the impact of predictive factors on the EFI risk in the optimal model, addressing the “black box” nature of ML models (20). SHAP provides global interpretability by ranking feature importance and revealing associations with EFI risk. This approach enhances model transparency, aiding clinical decision-making and personalized risk assessment.

This study adhered to the Transparent Reporting of a Multivariate Predictive Model for Individual Prognosis or Diagnosis (TRIPOD) guidelines and a step-by-step guideline for developing clinical prediction models (21, 22).

## 3 Results

### 3.1 Patient characteristics

A Following data screening and cleaning, 549 sepsis patients who met the inclusion criteria were included in the study. Of these, 190 cases (34.60%) developed EFI. Among the participants, 388 (70.7%) were male, and 161 (29.3%) were female, with ages ranging from 21 to 96 years and a mean age of 63.79 ± 14.80 years. The demographic and clinical characteristics of the non-EFI and EFI groups included in the model are presented in Table 1. In univariate analysis, 13 variables were found to be associated with the outcome (*p*-value < 0.05), and were subsequently included in the multivariate logistic regression analysis.

**Table 1:** Characteristics of sepsis patients with and without EFI in the model development dataset (*N* = 549).

Variable	No EFI group (*N* = 359)	EFI group (*N* = 190)	*χ*^2^/*Z*	*p*
Gender				
Male	261 (72.7%)	127 (66.8%)	2.058^a^	0.151
Female	98 (27.3%)	63 (33.2%)		
Combined chronic diseases				
No	139 (38.7%)	60 (31.6%)	2.741^a^	0.098
Yes	220 (61.3%)	130 (68.4%)		
Resistant bacterial infection				
No	291 (81.1%)	145 (76.73%)	1.710^a^	0.191
Yes	68 (18.9%)	45 (23.7%)		
Received CRRT treatment				
No	181 (50.4%)	65 (34.2%)	13.197^a^	< 0.001
Yes	178 (49.6%)	125 (65.8%)		
Mechanical ventilation				
No	118 (32.9%)	48 (25.3%)	3.407^a^	0.065
Yes	241 (67.1%)	142 (74.7%)		
Use of analgesics				
No	123 (34.3%)	57 (30.0%)	1.024^a^	0.312
Yes	236 (65.7%)	133 (70.0%)		
Use of sedatives				
No	112 (31.2%)	65 (34.2%)	0.516^a^	0.472
Yes	247 (68.8%)	125 (65.8%)		
Use of vasopressors				
No	129 (35.9%)	76 (40.0%)	0.878	0.349
Yes	230 (64.1%)	114 (60.0%)		
EN feeding methods				
Nasogastric tube	189 (52.6%)	102 (53.7%)	0.054^a^	0.817
Nasoenteric tube	170 (47.4%)	88 (46.3%)		
Type of nutritional formula				
Whole protein formula (A)	105 (29.2%)	77 (40.5%)	7.421^a^	0.006
Short peptide formula (B)	94 (26.2%)	37 (19.5%)		
A and B	131 (36.5%)	74 (38.9%)		
Others	29 (8.1%)	2 (1.1%)		
EN start time				
≤ 48 h	223 (62.1%)	140 (73.7%)	18.172^a^	< 0.001
> 48 h	136 (37.9%)	50 (26.3%)		
IAP				
< 12 mmHg	288 (80.2%)	64 (33.7%)	36.960^a^	< 0.001
≥ 12 mmHg	71 (19.8%)	126 (66.3%)		
Age (years)	66.00 (54.00, 72.25)	69.00 (60.50, 77.25)	−3.991^b^	< 0.001
BMI	23.93 (21.61, 24.57)	23.91 (21.12, 24.52)	−0.076^b^	0.940
Types of antibiotics	4.50 (2.00, 8.00)	6.00 (3.00, 8.00)	−1.759^b^	0.079
APACHE II score	17.00 (12.00, 20.00)	28.00 (24.00, 32.00)	−15.455^b^	< 0.001
NRS-2002 score	4.00 (3.00, 5.00)	4.50 (4.00, 5.00)	−0.629^b^	0.529
Blood glucose (mmol/L)	10.21 (8.02, 12.50)	10.12 (8.39, 12.10)	−0.911^b^	0.363
White blood cell count (10^9^/L)	11.67 (8.47, 14.62)	10.21 (8.02, 12.50)	−0.238^b^	0.812
Hemoglobin level (g/L)	89.00 (77.75, 100.25)	83.50 (74.38, 92.50)	−2.334^b^	0.020
Albumin (g/L)	32.74 (31.00, 34.30)	32.43 (30.43, 33.09)	−2.503^b^	0.012
Potassium (mmol/L)	3.96 (3.72, 4.13)	3.98 (3.74, 4.23)	−0.267^b^	0.790
Sodium (mmol/L)	142.28 (138.30, 144.00)	142.31 (138.10, 144.93)	−0.149^b^	0.882
Magnesium (mmol/L)	0.90 (0.83, 0.90)	0.91 (0.82, 0.95)	−0.029^b^	0.976
Procalcitonin (ng/mL)	2.59 (0.75, 10.76)	3.70 (1.50, 10.76)	−2.431^b^	0.015
Interleukin-6 (pg/mL)	116.07(34.82, 789.18)	150.19 (51.30, 769.18)	−2.517^b^	0.012
Lymphocyte percentage (%)	7.03 (4.40, 9.05)	5.30 (3.20, 8.20)	−3.718^b^	< 0.001
Monocyte percentage (%)	4.45 (3.10, 6.10)	3.53 (2.00, 5.10)	−3.203^b^	0.001
Lactate (mmol/L)	1.83 (1.30, 2.21)	1.82 (1.39, 2.30)	−2.226^b^	0.026

APACHE II, Acute Physiology and Chronic Health Evaluation II; EN, enteral nutrition; IAP, intra-abdominal pressure.

a Indicates the result of the chi-square test.

b Indicates the result of the non-parametric test.

### 3.2 Binary logistic regression analysis of factors influencing EFI in sepsis patients

Based on the results of the univariate analysis, 13 risk factors with *p* < 0.05 were selected as independent variables, including 9 continuous variables and 4 categorical variables. Categorical variables were assigned values, and continuous variables retained their original values, before being included in the multivariate logistic regression analysis. The results are detailed in Table 2.

**Table 2:** Results of logistic regression analysis on factors significantly (*N* = 549).

Variable	*β*	Standard Error	Wald	*p*	OR (95%CI)
Age	0.030	0.008	12.634	< 0.001	1.031(1.013–1.048)
APACHE II score	0.155	0.023	43.578	< 0.001	1.168(1.115–1.223)
Albumin	−0.076	0.028	6.991	0.008	0.926(0.875–0.980)
Received CRRT	1.396	0.252	30.702	< 0.001	0.958(0.932–0.985)
EN start time	1.016	0.250	16.450	< 0.001	2.764(1.691–4.517)
IAP	2.080	0.246	71.465	< 0.001	8.009(4.944–12.975)
Constant	−5.182	1.284	16.273	< 0.001	0.005

### 3.3 Model development

Ultimately, 6 variables were selected as predictive factors for model development: age, APACHE II score, albumin level, receipt of continuous renal replacement therapy, time of EN initiation, and IAP. A 7:3 stratified sampling method was used to divide the data into a training set (384 cases) and a validation set (165 cases). 5 ML models were constructed: decision tree, random forest, support vector machine (SVM), XGBoost, and neural network. Model performance was evaluated using 10-fold cross-validation and validation set testing.

### 3.4 Independent ML model performance and evaluation

**Figure 2:**
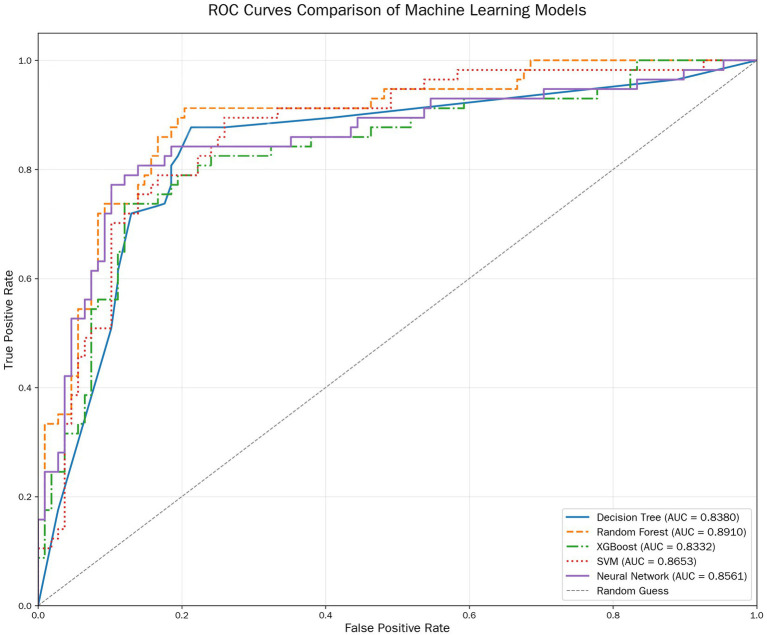
Receiver operating characteristic curves of score for 5 ML models. Curves illustrate the predictive performance of the 5 ML models in the validation cohort.

The confusion matrix results for each model show the detailed distribution of predictions (Supplementary Figure S1), with classification performance metrics for each model on the validation set presented in Supplementary Table S1. The random forest model performed the best, with an accuracy of 0.830, an F1 score of 0.771, specificity of 0.869, and sensitivity of 0.763, demonstrating strong overall classification ability. The support vector machine model followed closely, with an accuracy of 0.818, an F1 score of 0.727, and both specificity and sensitivity of 0.719, indicating stable classification performance. The XGBoost model achieved an accuracy of 0.806, an F1 score of 0.714, specificity of 0.842, and sensitivity of 0.719, excelling in identifying patients without EFI. The decision tree model had an accuracy of 0.733 and an F1 score of 0.714, with slightly lower performance across all metrics compared to the other models. Although the neural network model had a high specificity of 0.953, it showed a clear prediction bias towards certain classes and exhibited suboptimal overall performance. The overall performance of all 5 models during internal validation is detailed in Supplementary Table S1 and supported by the ROC curve (Figure 2). A 10-fold cross-validation (Supplementary Figure S2) was performed to further evaluate model stability, revealing minimal variation between folds, thereby supporting the reliability and stability of the predictive models.

### 3.5 Feature importance and model interpretation

**Figure 3:**
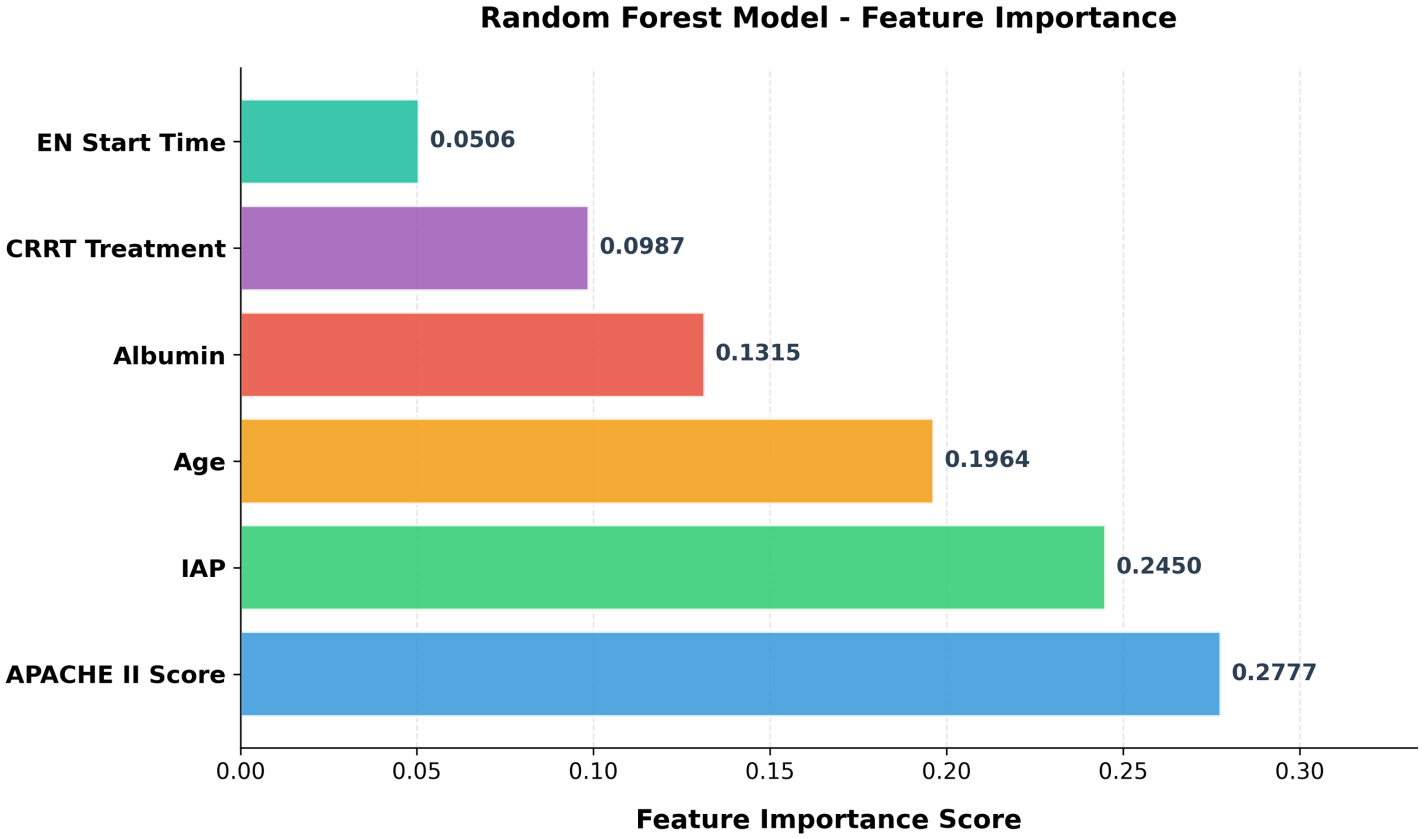
Feature importance of the random forest model. The 6 key predictive features for EFI are ranked in order of importance: APACHE II, IAP, age, albumin, CRRT treatment, EN start time.

**Figure 4:**
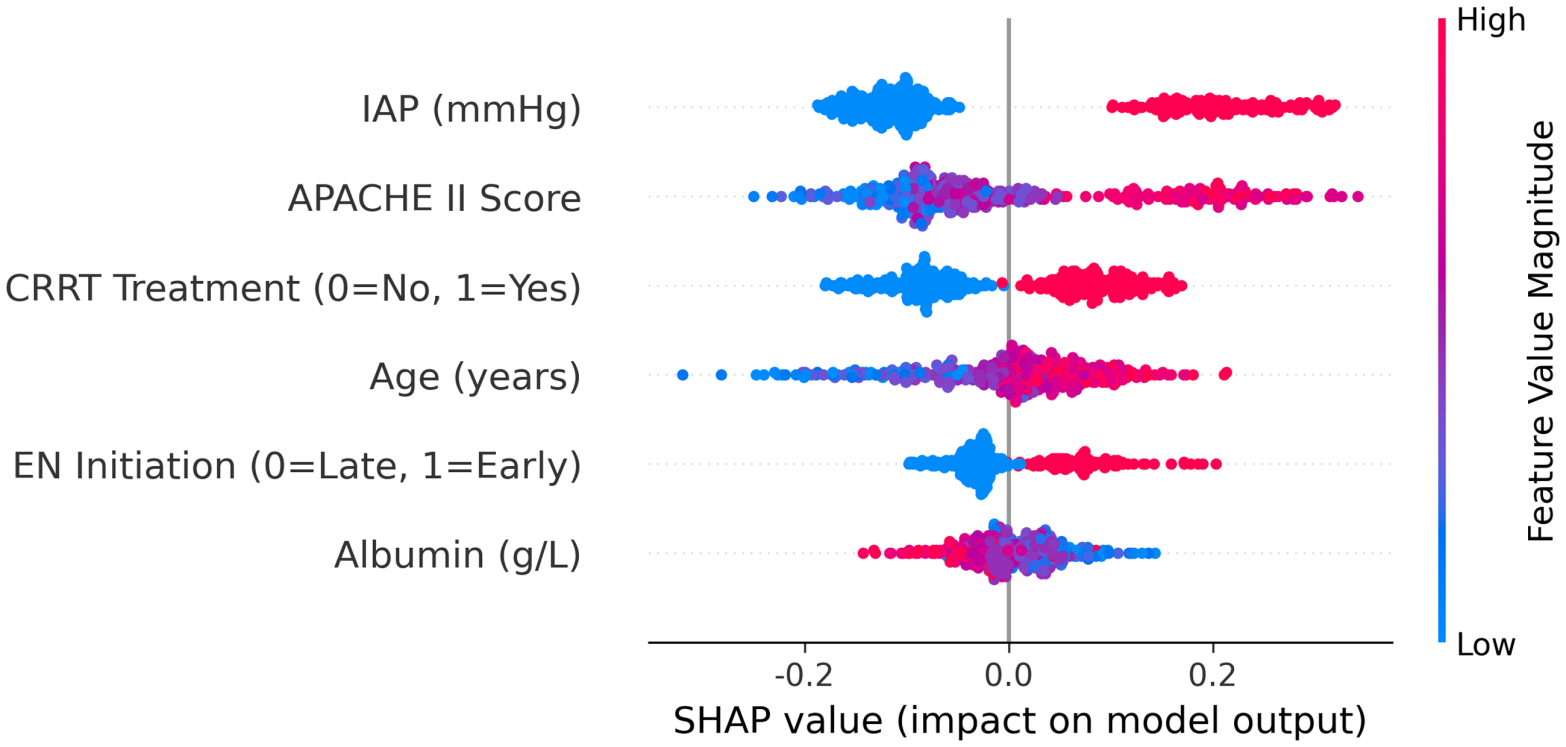
Dot plot of SHAP summary. The SHAP summary dot plot illustrates the impact of each feature on the risk of EFI. A higher SHAP value corresponds to an increased risk of EFI. Each dot represents the SHAP value for an individual patient, and dot color indicates feature magnitude (red = high value, blue = low value). Vertical dot density reflects the distribution of feature values.

Based on the optimal model (RF), a feature importance plot was constructed (Figure 3), which identified the top 6 factors influencing EFI risk: APACHE II score, followed by intra-abdominal pressure (IAP), albumin level, receipt of CRRT, age, and time of EN initiation. SHAP value analysis further revealed the direction and strength of the influence of each clinical feature on the model output (Figure 4). The SHAP values for APACHE II score and IAP were predominantly positive, indicating that higher levels of these indicators significantly increase the risk of EFI. The SHAP values for albumin level were mostly negative, suggesting that higher albumin levels are associated with a lower risk of EFI. Patients who received CRRT, were older, or had a later initiation of EN showed predominantly positive SHAP values, corresponding to an increased risk of EFI. SHAP analysis visually presented the quantifiable relationship between each feature and EFI risk, providing an interpretable basis for clinical risk assessment.

## 4 Discussion

EFI is a key factor contributing to disease progression and multi-organ failure in septic patients (23). Sepsis induces systemic inflammatory responses and multiple organ dysfunction, affecting both pulmonary and gastrointestinal function. (5, 24) Therefore, identifying EFI risk factors early is essential for prompt diagnosis and treatment. Our study included 549 samples, with 190 patients diagnosed with EFI, resulting in a 34.6% incidence rate, consistent with prior reports (12). This study addressed the need for EFI risk prediction in septic patients by developing 5 predictive models: decision tree, random forest, XGBoost, SVM, and neural networks. We evaluated model performance from multiple perspectives, selected the optimal model, and identified key risk indicators using feature importance analysis and SHAP value interpretation. This provides a quantitative tool and theoretical foundation for the clinical prevention and management of EN intolerance. APACHE II score, intra-abdominal pressure, and age are the primary factors influencing EFI, followed by albumin levels, CRRT treatment, and EN initiation timing. The contribution of each feature to the model output varied considerably.

This study shows that the Acute Physiology and Chronic Health Evaluation II (APACHE II) score is an effective predictor of EFI in septic patients. We found that as the APACHE II score increases, the risk of EFI rises significantly. This finding is consistent with existing literature, which suggests that the APACHE II score reflects sepsis severity and may also indirectly reflect gastrointestinal function (25, 26). High APACHE II scores are associated with dysfunction in multiple organ systems, including impaired gut blood flow, intestinal barrier function, and motility, all of which may reduce tolerance to enteral feeding (27, 28). These findings align with clinical cases, where severely ill septic patients show more pronounced gastrointestinal symptoms, such as bloating, nausea, vomiting, and diarrhea, during enteral feeding. These findings emphasize the need for caution when administering enteral feeding to critically ill septic patients, particularly those with high APACHE II scores. Early nutritional risk assessment and personalized nutrition support are critical for these patients. Since the APACHE II score assesses illness severity in critically ill patients, clinicians may need to gradually increase enteral feeding or switch to parenteral nutrition if enteral feeding is not tolerated, to optimize nutrition and reduce complications.

This study identifies age as a major risk factor for EN intolerance in sepsis patients, consistent with findings from previous studies (29, 30). As individuals age, physiological functions, particularly those of the immune, digestive, and metabolic systems, decline, making elderly patients more prone to EN intolerance (31). Elderly patients often suffer from impaired gut barrier function and dysbiosis, leading to inefficient nutrient absorption and utilization, which in turn triggers intolerance reactions (32). Additionally, older patients often have chronic conditions such as hypertension, diabetes, and cardiovascular diseases, which can impair gut function and metabolism, further raising the risk of EN intolerance (33). Reduced gut motility in elderly patients can lead to prolonged retention of EN, increasing intestinal burden and exacerbating symptoms of intolerance (34). In clinical practice, special attention should be given to the tolerance of EN in older sepsis patients, especially those with multiple comorbidities. Age should be included in risk prediction models to identify high-risk patients early, facilitating timely adjustments to nutritional support and preventing intolerance. Personalized nutritional interventions can greatly improve the nutritional status and clinical outcomes of elderly sepsis patients.

Our study identified intra-abdominal pressure (IAP) as a significant predictor of EFI in septic patients, consistent with recent research findings (35). IAP is the pressure within the abdominal cavity under normal conditions. In critically ill patients, it fluctuates between 5–7 mmHg, with minor variations due to physiological activities such as breathing. A pressure exceeding 12 mmHg is classified as intra-abdominal hypertension, which, if sustained, can negatively impact systemic circulation, liver and renal blood flow, and respiratory function (36). In sepsis, uncontrolled systemic inflammation, increased capillary leakage, and fluid resuscitation commonly result in intestinal edema, ascites, and gastrointestinal motility dysfunction, leading to intra-abdominal hypertension (37, 38). This study employed bladder pressure measurement, the clinical gold standard, to quantify IAP, ensuring data reliability. Intra-abdominal hypertension impacts gut function through several pathophysiological mechanisms. First, it directly compresses mesenteric vessels, reducing intestinal blood flow and worsening the pre-existing microcirculatory dysfunction in sepsis, leading to intestinal mucosal ischemia and hypoxia (38). Second, direct mechanical compression of the intestinal wall reduces gut compliance, impedes the passage of contents, and suppresses gastrointestinal motility (39). These mechanisms together lead to typical symptoms of EFI, including increased gastric residuals, bloating, and vomiting. Therefore, incorporating IAP—an objective, measurable physiological indicator—into risk prediction models is grounded in strong pathophysiological evidence and provides crucial support for the early identification of high-risk patients and personalized interventions.

This study identifies albumin levels as a significant predictor of EFI in sepsis patients. Albumin is a crucial biomarker that reflects systemic nutritional status and inflammatory response. In sepsis patients, its levels are often markedly reduced, correlating strongly with disease severity, organ dysfunction, and poor prognosis (40). Previous studies have demonstrated that low albumin levels are associated not only with poor nutritional status but also with an increased risk of EN intolerance, due to their effects on gut blood flow, barrier function, and microcirculation (41). In sepsis, decreased albumin levels can compromise intestinal barrier function, resulting in gut ischemia, hypoxia, and worsened gastrointestinal inflammation, and this suppression of intestinal motility subsequently increases the incidence of EN intolerance (42). Our study, supported by the SHAP summary plot, confirms that albumin serves as a protective factor in sepsis, consistent with previous research findings (43). One study indicates (44) that hypoalbuminemia is a significant risk factor for enteral nutrition-associated diarrhea in critically ill patients, possibly due to decreased plasma colloid osmotic pressure, leading to intestinal mucosal edema and impaired absorption. Mizui et al. (45) confirmed that serum albumin levels below 2.5 g/L cause intestinal wall mucosal edema, and levels below 1.5 g/L result in a 100% incidence of diarrhea. Sepsis patients with hypoalbuminemia upon admission should be closely monitored for EN intolerance risk. Proactive preventive strategies, such as conservative feeding protocols, enhanced gastrointestinal symptom monitoring, or early combined parenteral nutrition support, should be implemented to manage the acute phase safely and improve clinical outcomes.

The SHAP analysis offers a clear visual representation of the relationship between each feature and the risk of EFI, providing an interpretable basis for clinical risk assessment. For patients receiving CRRT or starting EN later, SHAP values are generally positive, indicating a higher risk of EFI. The 2017 expert guidelines recommend that most critically ill patients begin EN within 48 h, instead of receiving total or supplemental parenteral nutrition alongside early EN (46). A meta-analysis showed that patients receiving EN within 24–48 h of ICU admission tend to have shorter mechanical ventilation durations and lower rates of infection and EFI (47). Therefore, septic patients should receive EN support within 48 h of ICU admission to restore gastrointestinal function. CRRT is the primary form of renal replacement therapy in the ICU, ensuring accurate volume control, stable acid–base and electrolyte correction, and supporting hemodynamic stability in patients with severe acute kidney injury, chronic kidney disease, and sepsis (48). However, our study found that patients undergoing CRRT face greater challenges in achieving target EN. First, CRRT itself is a major stressor, indicating more critical conditions, often accompanied by severe systemic inflammation and multi-organ dysfunction, making it an inherent high-risk factor for feeding intolerance (49). More critically, hemodynamic fluctuations associated with CRRT, combined with sedation and analgesia treatments, suppress gastrointestinal motility, directly hindering the implementation of EN (43, 50). Therefore, our findings suggest that clinicians should not consider CRRT as a “green light” for early EN implementation. Rather, it should signal the need for more proactive and refined EN management. As noted, CRRT and early EN are interconnected and play a significant role in the metabolic support of critically ill patients. Future guidelines and clinical practices should focus on developing personalized nutritional strategies for this population to break the cycle of critical illness and malnutrition.

In this study has several limitations, which should be addressed in future research. First, the predictive model’s development and validation relied mainly on retrospective data from a single-center cohort, rather than a multi-center, prospective cohort. This may introduce bias and limit its generalizability. Second, the model used laboratory values collected within 24 h at a single time point, which may not reflect the dynamic changes in a patient’s condition. Third, due to sample size and time constraints, external validation was not conducted, potentially limiting the model’s generalizability and clinical applicability across different patient populations. Finally, the model has not yet been implemented in clinical practice via web-based or hospital information system (HIS)-based tools, which may hinder its future application.

## 5 Conclusion

In conclusion, we developed an interpretable ML model to predict EFI in septic patients. The final RF model demonstrated high predictive performance and assists in the early identification, prevention, and management of EFI in septic patients. Future research should prioritize external validation using multi-center cohort data and the development of web-based or HIS-integrated applications to enhance clinical accessibility. Additionally, a mobile app or mini-program could be developed to support early screening of EFI, facilitating early intervention and prevention. Simultaneously, this model could be developed into a scale-based assessment tool for clinical use, enabling its scientific validity and feasibility to be tested in practice and promoting knowledge translation and clinical application. Future randomized controlled trials are needed to evaluate whether personalized, timely treatment interventions based on the proposed model can improve patient outcomes.

## Data Availability

The original contributions presented in the study are included in the article/Supplementary material, further inquiries can be directed to the corresponding authors.
