# Supplementary material for: Development and evaluation of a machine learning-based risk prediction model for enteral feeding intolerance in sepsis patients
Source: Front Nutr. 2026 Jun 10;13:1858783. doi: 10.3389/fnut.2026.1858783 (PMC13290633; doi:10.3389/fnut.2026.1858783)
Supplement: Supplementary file 1 [file Table_1.docx]

Supplementary Material

# Supplementary Figures and Tables

## Supplementary Figures


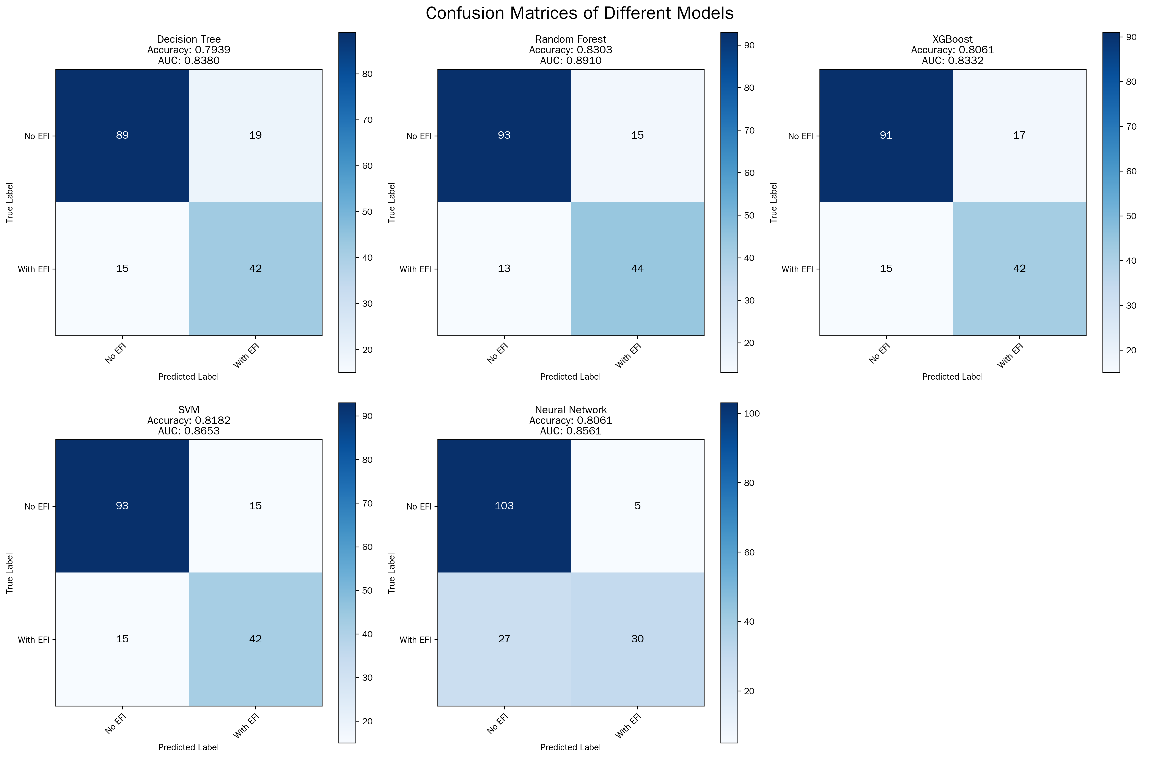


**FIGURE S1**

Confusion Matrix of Internal Validation. Confusion matrices illustrate the classification performance of the five machine learning models in the validation cohort.


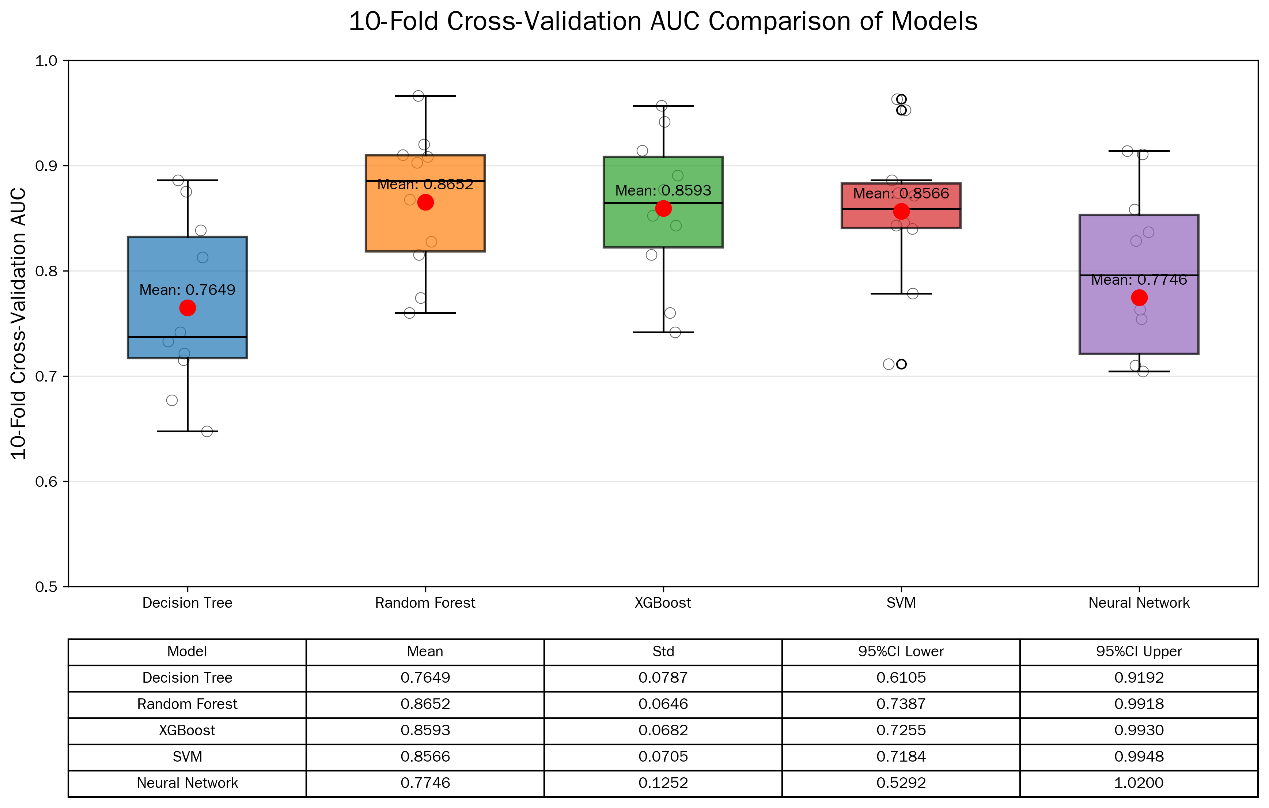


**FIGURE S2**

The performance of 10-Fold Cross-Validation for 12 ML models. 10-fold cross-validation was performed to validate the stability and reliability of the random forest model.

## Supplementary Tables

**TABLE S1** Overall performance for the five ML models in internal validation dataset.

| **Model** | **Accuracy** | **F1** | **Specificity** | **Sensitivity** |
| --- | --- | --- | --- | --- |
| Decision Tree | 0.733 | 0.714 | 0.824 | 0.736 |
| Random Forest | 0.830 | 0.771 | 0.869 | 0.763 |
| XGBoost | 0.806 | 0.714 | 0.842 | 0.719 |
| SVM | 0.818 | 0.727 | 0.854 | 0.719 |
| Neural Network | 0.806 | 0.530 | 0.953 | 0.526 |
